# Optical mapping in plant comparative genomics

**DOI:** 10.1186/s13742-015-0044-y

**Published:** 2015-02-10

**Authors:** Haibao Tang, Eric Lyons, Christopher D Town

**Affiliations:** 1 Center for Genomics and Biotechnology, Fujian Agriculture and Forestry University, Fuzhou, 350002, Fujian People's Republic of China; 2 School of Plant Sciences, iPlant Collaborative, University of Arizona, Tucson, AZ 85721 USA; 3 J. Craig Venter Institute, Rockville, MD 20850 USA

**Keywords:** Optical mapping, Comparative genomics, *De novo* assembly, Structural variation

## Abstract

Optical mapping has been widely used to improve *de novo* plant genome assemblies, including rice, maize, Medicago, Amborella, tomato and wheat, with more genomes in the pipeline. Optical mapping provides long-range information of the genome and can more easily identify large structural variations. The ability of optical mapping to assay long single DNA molecules nicely complements short-read sequencing which is more suitable for the identification of small and short-range variants. Direct use of optical mapping to study population-level genetic diversity is currently limited to microbial strain typing and human diversity studies. Nonetheless, optical mapping shows great promise in the study of plant trait development, domestication and polyploid evolution. Here we review the current applications and future prospects of optical mapping in the field of plant comparative genomics.

## Introduction

Optical mapping is a molecular technique that produces fingerprints of DNA sequences in order to construct genome-wide maps [1]. The sequence markers can be ordered restriction fragments [1], or specific sequence motifs (nick sites) [2]. The optical mapping procedure first stretches relatively intact (minimally-sheared) linear DNA fragments on a glass surface or in a nanochannel array, and then directly images the locations of the restriction sites or sequence motifs under light microscopes, with the aid of dye or fluorescent labels [1,2]. Automation of optical measuring and processing devices has led to the development of commercial platforms, such as OpGen Argus [3] and BioNano Genomics Irys systems [4].

Optical mapping offers several unique advantages over traditional mapping approaches, including single molecule analysis and the ability to assay long DNA molecules (~250Kb to 3 Mb in conventional optical mapping [1] and 20-220Kb in nanochannel arrays [2]). The ability to assay large DNA molecules has allowed accurate reconstruction of chromosomal pieces during *de novo* genome assembly and identification of relatively large structural variants in genetic diversity studies. While optical mapping is readily available across a wide range of organisms including bacterial, fungi, plant and mammalian genomes [5-9], this review focuses on the applications and of optical mapping in the field of plant comparative genomics.

## Review

### Optical map guided genome assemblies

A hierarchical approach is typically adopted for building a high quality genome assembly for most organisms – starting with identifying read overlaps to build contigs, then adding read pairs to build scaffolds, and finally ordering scaffolds to assemble large chromosomal regions using various sources of long distance mapping information [10]. There are several ways in the assembly process that optical mapping can assist in building high quality reference genomes. *De novo* constructed optical maps offer independent evidence to connect and bridge adjacent sequence contigs or scaffolds [6,11]. Optical maps can also suggest potential errors in the scaffold assembly [11,12]. Additionally, it is also possible to directly exploit optical map information during genome assembly to help determine the correct path through the assembly graph [13].

Genome assemblies guided by optical maps consist of three key computational steps. The initial step is the *de novo* assembly of optically mapped molecules to construct a ‘consensus’ optical map from single DNA molecules at high redundancy. The consensus map has to deal with errors specific to optical mapping including missing cuts, false cuts, inaccurate fragment sizes, and chimeric maps [14]. The next step is to align the *in silico* digested contig sequences to the consensus optical map [15,16]. The final step is the joining of neighboring contig sequences to construct supercontigs on the basis of their locations on the optical map [8]. For small microbial genomes, the resulting assemblies could contain a single extent of sequence that spans the entire genome [8], while for large eukaryotic genomes the combined efforts of sequencing and optical mapping often result in substantially increased scaffold *N50* (Table 1). In several cases, the mapping data allow the reconstruction of entire chromosomes [11,17].

**Table 1:** **Published plant studies utilizing optical mapping for the improvement of** ***de novo*** **genome assemblies**

Organism	Sequence size	Details of improvement	Reference
*Oryza sativa*	373 Mb	Corrected 23 potential errors in the BAC tiling path	Kawahara *et al.* 2013 [18]
*Zea Mays*	2,061 Mb	Placed 60/66 FPC contigs; Replaced 12 FPC contigs	Zhou *et al.* 2009 [12]
*Medicago truncatula*	412 Mb	Scaffold *N50* improved from 4.2 Mb to 49.2 Mb (8 pseudomolecules); Evidence used in genome version Mt4.0	Tang *et al.* 2014 [11]
*Amborella trichocarpa*	706 Mb	Scaffold *N50* improved from 4.9 Mb to 9.3 Mb	Chamala *et al.* 2013 [6]
*Prunus mume*	237 Mb	Scaffold *N50* improved from 578Kb to 1.1 Mb	Zhang *et al.* 2012 [18]
*Solanum lycopersicum*	760 Mb	Fully compatible with FISH results but suggested only 22/38 compatible with linkage map; Evidence used in genome release SL2.5	Shearer *et al.* 2014 [20]
*Aegilops tauschii*	2.1 Mb	Sequence completeness improved from 75% to 95%	Hastie *et al.* 2013 [21]

BAC, Bacterial-artificial chromosome; FISH, Fluorescent *in situ* hybridization; FPC, Fingerprinted contig.

**Figure 1:**
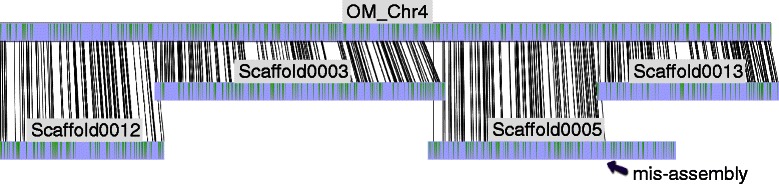
**Use of optical mapping in the*Medicago truncatula*genome assembly Mt4.0.** Scaffolds assembled from next-generation sequencing were aligned against the Medicago optical map (OM_Chr4) in order to build a pseudo-chromosome. Scaffold0005 was identified as a chimeric scaffold with its left part aligned to the optical map while the right part aligned to a different chromosome. Optical mapping provided an independent line of evidence to join adjacent scaffolds and split misassembled sequences during the reconstruction of chromosomal-sized sequences.

Beyond ordering and orientating contigs, optical maps provide an additional layer of validation to the sequence assemblies. Optical maps could potentially identify and resolve misassemblies – false joins, inversions or translocations that are artifacts, which occurred during the sequence assembly. Sequence scaffolds could be chimeric due to the reads residing in the repetitive regions of the genome. Consequently, chimeric scaffolds that align partially, or align to multiple distinct locations of the optical maps are suspect of misassemblies (Figure 1). The sites of potential sequence misassemblies could gain further support if the same ‘breakpoints’ are also indicated from other lines of evidence, such as genetic maps or physical maps [11].

Similar to optical maps, genetic maps could be a useful guide in anchoring scaffolds and identifying assembly issues [11,19]. However, recent studies suggested that genetic maps might be subject to errors, potentially confounding genome assemblies. Specific genomic structural features may cause issues during the construction of genetic maps, including chromosomal inversion, translocation, and segmental duplication that vary between the two parents used to generate the mapping population. Studies in tomato show that optical mapping and fluorescent *in situ* hybridization (FISH) data support each other, but are both inconsistent with linkage maps, especially in heterochromatic regions where recombination is scarce [20]. Similar discrepancies were discovered during the Medicago genome assembly, where a reciprocal translocation occurred between chromosome 4 and 8 in strain A17 (the reference genome), but was absent from the parents of the LR4 mapping population used to generate the linkage map [11]. Genome assemblies that rely solely on linkage maps could become unreliable due to unknown mapping errors, as well as structural differences in the individuals used to generate the linkage maps. These errors could be corrected by consulting the optical maps [11,20].

### Towards building ‘platinum’ quality reference genomes

Genome ‘upgrades’ , or improvement of genome assemblies are possible through the incorporation of the optical mapping information into existing sequences. For example, optical mapping was essential in upgrading the rice Nipponbare reference genome in several important ways [17,18]. First, optical mapping validated and corrected the Minimum Tiling Path (MTP) of bacterial artificial chromosomes (BACs) that were used to generate the reference genome. Second, the alignments between the sequenced BACs, P1-derived artificial chromosomes (PACs), pseudomolecules and the optical maps were manually examined to confirm concordance, and discordant regions were adjusted accordingly. Lastly, optical mapping facilitated the estimation of gap sizes by summing the length of un-aligned restriction fragments, which also identified the location of the physical gaps generated in highly repetitive centromeric or telomeric regions. The modifications employed in the rice genome upgrade, enabled by the use of optical mapping data, resulted in an estimated ~97% coverage of the entire rice genome [18].

Similar to rice, optical maps have been extensively used to improve the Medicago genome assembly starting with release version Mt3.5, and were helpful both during the chromosomal anchoring and to correct errors in the linkage maps [11,22]. To build an upgraded version of the Medicago reference genome (Mt4.0), sequences from a whole genome shotgun assembly and individually sequenced BACs were ordered and oriented based on the optical maps to construct a high quality genome release [11]. A total of 85.7% of the Mt4.0 assembly could be aligned to the Medicago optical maps, yielding a much improved assembly over the previous release [11]. During the assembly, optical map alignments suggested eleven breakpoints within chimeric scaffolds that aligned to disjoint regions of the genome (one example on chromosome 4 is shown in Figure 1). Among these, 9 breakpoints were also supported by genetic maps [11]. The high quality Medicago reference genome was shown to be valuable in legume comparative genomics. For example, the number of gene pairs derived from the papilionoid whole-genome duplication (WGD) inferred based on the Mt4.0 assembly nearly tripled the number of pairs identified in the previous version due to the much higher contiguity of the genome [11].

Optical mapping can be very useful in assisting the assembly of polyploid and highly heterozygous plant genomes, which are notoriously difficult to assemble [10]. Many plant genomes are especially abundant in repeats and high copy DNA elements that tend to stall short read assembly. Long DNA molecules of several hundred Kb can comfortably span most types of proximal or interspersed repeats so that they become less problematic for optical mapping. In polyploids, sequences from the co-resident subgenomes (especially if recently diverged) tend to confuse assembly algorithms that depend only on the short overlaps between reads. These subgenomes could have a better chance of separation based on long range optical mapping that more easily differentiates subgenomes of varying size or with different transposon composition.

### Comparisons of different approaches to identify structural variations

**Figure 2:**
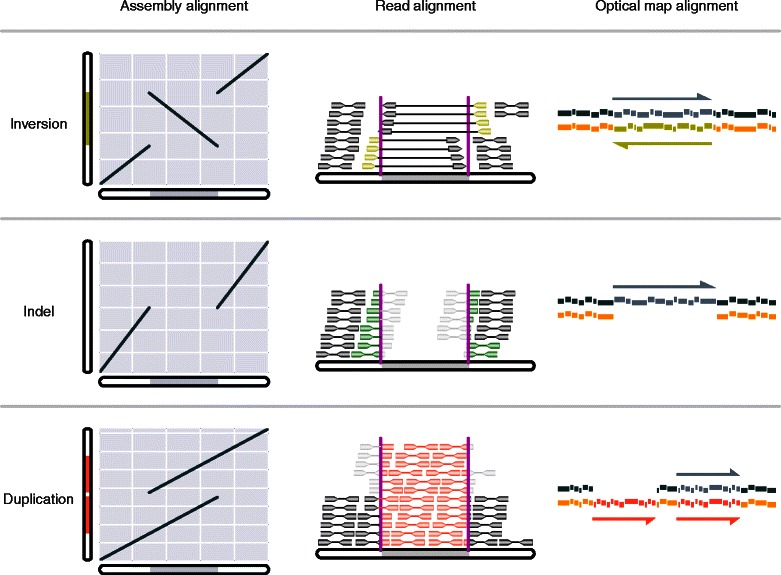
**Common types of genomic structural variations (SVs) detected based on the comparison of assembly, read alignments or optical maps, each relying on their unique ‘signatures’.** Alignment between assemblies reveals SVs through the disruption of otherwise collinear patterns of sequence matches. Alignment of reads against a reference genome reveals SVs through split reads, discordant read pairs, and aberrant read depth. Alignment between optical maps reveals SVs through the inverted, missing or extra fragment patterns.

Despite recent progress in genome assembly methodologies, a significant portion of many genomes remains inaccessible to assembly by short sequencing reads [10]. A comprehensive catalog of all genetic variants through sequencing, chips, mapping, or cytogenetic approaches reduces systematic bias associated with any single platform [7]. However, many classes of structural variants (SVs), including inversion, deletion, insertion, duplication and translocation, are under-explored due to the challenges in their accurate identification and subsequent tedious validation. Common SVs can be identified based on the comparisons of assembly, reads or optical maps, each with their respective advantages and disadvantages (Figure 2).

Pairwise sequence alignments between assembled genomes remains one of the most powerful tools for plant comparative genomics, and could identify SVs with the best accuracy if the assemblies themselves were correctly reconstructed. However, *de novo* assembly is still challenging and large amounts of true SVs may be lost during the assembly process. For most organisms, the ‘reference’ genome only represents a single individual and requires substantial amount of investment for the initial genome assembly and subsequent finishing. Most assemblies can only reach ‘draft’ status, often containing a large number of sequence gaps and assembly errors that could easily show up as false SVs during sequence comparisons.

High throughput re-sequencing uncovers various genetic variations by mapping the sequencing reads of related individuals to the reference genome. Aberrant alignment patterns could reveal SVs through several distinct signatures. *Split reads* at the same position could indicate genomic breakpoints derived from inversions, deletions and insertions. *Discordant pairs* reveal spacing difference due to deletions or insertions, or presence-absence variations (PAVs). *Read depth variations* can be used to identify copy number variations (CNVs) that are likely derived from tandem or segmental duplications (Figure 2). While highly effective towards single nucleotide substitutions and small indels, re-sequencing typically misses a considerable number of SVs at longer range due to the lack of long-range linkage information inherent in the short DNA fragment libraries. In this regard, longer insert mate pair libraries may be more informative. Additionally, many SVs are located in repetitive regions of the genome, where read mapping has a generally low sensitivity [9].

By comparison, direct alignments between optical maps provide a complementary view of the genetic variations between individuals, and differ from other comparative techniques in that fragment patterns, instead of nucleotide matches, form the basis of the alignments (Figure 2). Optical mapping provides linkage information that is otherwise not attainable from short reads, and can predict large SVs more easily than the read-mapping method. Direct optical map comparisons are routinely conducted in microbes [5] and in human [7], but have been lacking in the application to plant genomes until recently due to relatively high historical cost. However, these costs are falling due to rapid commercial development [3,4]. Applications of optical maps among cultivars or in natural populations would allow direct assessment of large structural changes that are several hundred Kb to Mb in size.

### Structural variations affecting plant traits

Local adaptation of plant varieties is reflected in traits, such as flower development, photo-sensitivity, disease resistance and stress tolerance. All of these traits have been shown to be associated with SVs in various taxa [23,24]. Some SVs may have been under intense natural and/or artificial selection [23]. For example, the *PROG1* gene was found to be deleted in several rice species, leading to prostrate rather than erect growth [24] that differentiates rice species. Due to the limitations of sequencing-based approaches, the impact of SVs on the diversification of plant varieties may still be under-estimated, but could be clarified via optical mapping.

Some important agronomic traits are directly caused by structural variations which could be studied with a whole genome association framework across varieties or diversity panels. For example, the *SUN* gene that controls elongated fruit shape of tomato results from long-terminal repeat (LTR) retrotransposon-mediated gene duplication [25]. Current studies mostly focus on single nucleotide polymorphisms (SNPs) or short indels as markers of association genetics, but have largely ignored the large SVs which often have significant genomic and functional impact. With the recent decrease in cost, we could conduct optical mapping on genetic mutants and re-sequencing lines to directly identify those critical SVs that are linked to the varietal differences.

### Optical mapping in an evolutionary framework

In addition to agronomic traits, a wide range of studies in plants, including domestication, polyploidy, population history and natural selection could benefit from optical mapping. Long *et al.* uncovered large structural variants that are associated with selective sweeps in Arabidopsis lines from Sweden, based on a suite of methods from ‘manual’ detection of breakpoints to *de novo* assembly. They acknowledged that many polymorphisms may be complex and difficult to resolve using short-read sequencing data [23]. Re-sequencing studies have also revealed that SVs in the maize genome are particularly enriched in regions important for domestication [26], although many candidate SVs remain to be validated using an independent approach, such as optical mapping.

The application of optical mapping could reveal structural changes following polyploidy events in plants that might be difficult to study using other techniques. Studies show that homeologous exchanges (HEs) occur frequently between subgenomes inside polyploid genomes and often involve large chromosomal segments. This was studied in the *Brassica napus* genome, an allotetraploid merged from two diploid *Brassica* genomes [27]. Each HE was characterized by the replacement of a particular region with a duplicated copy from another subgenome. Specific HEs have contributed to the deletions of genes responsible for glucosinolate catabolism, probably selected as a result of intense breeding [27]. While read mapping provided the initial clues about HEs, the precise locations and boundaries of HEs across a set of lines were difficult to assess based on read mapping, thereby requiring a tedious validation procedure based on PCR and targeted sequencing in the study reported [27]. The direct application of optical mapping could therefore help pinpoint the precise breakpoint and further validate segmental loss and exchanges among homeologous chromosomes, which are important aspects of polyploid genome evolution.

## Conclusions

Optical mapping is an important technique that can provide long genomic linkage information in a high-throughput manner, which has substantially improved the assemblies of several important model plant genomes sequenced to date. Direct comparisons of genome structures have so far been lacking in plants, but optical mapping shows great promises at revealing genomic regions that are not easily accessible through conventional sequencing methods. Optical mapping could become an integral part of the mapping tools in the study of plant domestication, polyploid evolution, and trait development.

## Abbreviations

BAC: Bacterial artificial chromosome
CNV: Copy number variation
FISH: Fluorescent *in situ* hybridization
FPC: Fingerprinted contigs
HE: Homeologous exchange
LTR: Long-terminal repeat
MTP: Minimum Tiling Path
PAC: P1-derived artificial chromosome
PAV: Presence-absence variation
SV: Structural variation
WGD: Whole-genome duplication
